# Long Non-coding RNA HIX003209 Promotes Inflammation by Sponging miR-6089 via TLR4/NF-κB Signaling Pathway in Rheumatoid Arthritis

**DOI:** 10.3389/fimmu.2019.02218

**Published:** 2019-09-18

**Authors:** Shushan Yan, Pingping Wang, Jinghua Wang, Jinghan Yang, Hongying Lu, Chengwen Jin, Min Cheng, Donghua Xu

**Affiliations:** ^1^Clinical Medicine College, Weifang Medical University, Weifang, China; ^2^Department of Gastrointestinal and Anal Diseases Surgery, The Affiliated Hospital of Weifang Medical University, Weifang, China; ^3^Department of Gynecology and Obstetrics, Weifang Hospital of Maternal and Child Health, Weifang, China; ^4^Department of Rheumatology, The Affiliated Hospital of Weifang Medical University, Weifang, China; ^5^Functional Laboratory, Clinical Medicine College of Weifang Medical University, Weifang, China; ^6^Department of Physiology, Weifang Medical University, Weifang, China

**Keywords:** long non-coding RNA, competitive endogenous RNA, miRNA, inflammation, toll-like receptor, NF-κB

## Abstract

Accumulating studies have suggested that long non-coding RNAs (lncRNAs) have drawn more and more attention in rheumatoid arthritis (RA), which can function as competitive endogenous RNAs (ceRNAs) in inflammation and immune disorders. Previously, we have found that lncRNA HIX003209 is differentially expressed in RA. However, the precise mechanism of lncRNA HIX003209 in RA is still vague. We aim to elucidate the role and its targeted microRNA of lncRNA HIX003209 in RA as ceRNA. Significantly increased expression of lncRNA HIX003209 was observed in the peripheral blood mononuclear cells (PBMCs) from RA cases. It was positively associated with TLR2 and TLR4 in RA. Besides, peptidoglycan (PGN) and lipopolysaccharide (LPS) could enhance the expression of lncRNA HIX003209, which reversely promoted the proliferation and activation of macrophages through IκBα/NF-κB signaling pathway. Moreover, HIX003209 was involved in TLR4-mediated inflammation via targeting miR-6089 in macrophages. LncRNA HIX003209 functions as a ceRNA and exaggerates inflammation by sponging miR-6089 through TLR4/NF-κB pathway in macrophages, which offers promising therapeutic strategies for RA.

## Introduction

Rheumatoid arthritis (RA) is a systemic autoimmune disease, the etiology of which remains largely unknown (1, 2). RA Patients usually have decreased quality of life due to progressive disability and systemic complications (3, 4). It has been well-documented that genetics and environmental factors, such as smoking, are associated with the development of RA (4). Apart from autoimmune, uncontrolled and systemic inflammation lead to joint damage, disability, decreased life quality, and increased risk of cardiovascular comorbidities among RA patients. Accordingly, it is essential to explore molecular mechanisms involved in inflammation in order to explore novel potential therapeutic strategy for RA.

Accumulating studies have suggested that non-coding RNAs, particularly long non-coding RNAs (lncRNAs), have been revealed in inflammation, cancer and autoimmune (5, 6). LncRNAs can crosstalk with immune cells and mediate immunological and inflammatory response through nuclear factor-κB (NF-κB) signaling pathway (7–10). Recent studies have implicated a number of dysregulated lncRNAs contribute to the inflammatory response in RA (8, 11). Certain differentially expressed lncRNAs in RA have been demonstrated to affect the disease activity (12). Increasing evidence has revealed lncRNAs may regulate microRNAs (miRNAs) via functioning as competitive endogenous RNAs (ceRNAs), and thus participate in autoimmune diseases, including RA (13, 14). It is well-known that miRNAs can cause gene silencing by binding to mRNAs, while lncRNAs are capable of promoting the expression of targeted mRNAs by sponging miRNAs through the response element. Therefore, the lncRNA-miRNA-mRNA network possesses great significance in various biological processes. However, little has been known about the altering effect of lncRNA-miRNA-mRNA network in RA up till now. Previously, we have identified a novel lncRNA HIX003209 up-regulated in RA patients by microarray analysis (15). Nevertheless, the precise role and mechanisms of lncRNA HIX003209 in RA pathogenesis remain unclear, particularly regarding its role as a ceRNA in regulating inflammation and autoimmunity. The object of the study is to explore the role and molecular mechanisms of lncRNA HIX003209 in RA.

## Materials and Methods

### Participants

RA patients (76) and age and sex-matched controls (60) were recruited from the hospital at the same period. Controls came to the same hospital for health examination. There was no difference for the status of ethnicity, smoking, alcohol consumption, and citizens of origin between the two groups. Table 1 showed detailed information about the characteristics of all participants. Written informed consent was obtained from all participants before blood samples preparation. The study was permitted by the ethical committees in the Affiliated Hospital of Weifang Medical University.

**Table 1:** Characteristics of patients and controls.

	**RA**	**Controls**
Age (mean ± SD)	57.9 ± 20.1	55.3 ± 19.8
Sex (women/man)	50/26	40/20
Smoking (years)	28.9 ± 10.1	27.0 ± 11.3
Alcohol (years)	19.8 ± 8.5	15.2 ± 6.9
CRP (mg/L)	41.2 ± 15.9	5.0 ± 5.1
ESR (mm/h)	58.4 ± 17.6	13.1 ± 9.0
RF (IU/ml)	161.5 ± 45.4	14.6 ±6.3

### Cell Culture and Transfection

THP-1 cells were cultured in RPMI 1640 (Invitrogen, USA) adding 10% fetal bovine serum (Gibco, USA) in company with penicillin/streptomycin (Invitrogen, USA). Firstly, THP-1 Cells were induced to be macrophages-like cells (pTHP-1) by 100 nM phorbol-12-myristate-13 acetate (PMA, Sigma, USA). Cells were activated by PMA for 48 h. After being cultured in fetal bovine serum-free serum for another 24 h, pTHP-1 cells were transfected by lentivirus particles in accompany with polybrene reagent. Peripheral blood mononuclear cells (PBMCs) of all participants were purified by Ficoll-Paque gradient centrifugation. CD14^+^ mononuclear macrophages were separated by use of the CD14 microbeads (Miltenyi Biotec, San Diego, CA) according to the instructions.

### Real-Time Polymerase Chain Reaction (PCR)

Based on the instructions of Trizol reagent (Invitrogen, CA, USA), RNAs were isolated from human PBMCs, primary macrophages, or cell lines. A total of 0.5 μg RNAs were used as model for the synthesis of cDNAs. We used the Takara SYBR Green Mastermix kit (Tianjin, China) for PCR with a total of 5 ng cDNAs as template. The relative expression of TLR2, IL-6, TLR4, TNF-α, and IL-8 mRNAs was normalized to GAPDH. Genes primers were as follows: TNF-α: (F): 5′~3′ GTCAACCTCCTCTCTGCCAT, (R): 5′~3′ CCAAAGTAGACCTGCCCAGA; HIX003209, (F): 5′~3′ ACTGCTCGCCAGAACACTAC, (R): 5′~3′ GGTGAGGTTGATCGGGGTTT; IL-6, (F): 5′~3′ AGTCCTGATCCAGTTCCTGC, (R): 5′~3′ CTACATTTGCCGAAGAGCCC; IL-8: (F): 5′~3′, CGGAAGGAACCATCTCACTG, (R): 5′~3′ TTGGGGTGGAAAGGTTTGGA; TLR2: (F): 5′~3′, CTATGAATCAAGGCGGCCAC, (R): 5′~3′, AAAGATCCTGAGCTGCCCTT; TLR4: (F): 5′~3′ CCAGCCTCCTCAGAAACAGA, (R): 5′~3′ TCCCTCCAGCAGTGAAGAAG; GAPDH: (F): 5′~3′ CTGACTTCAACAGCGACACC, (R): 5′~3′ GTGGTCCAGGGGTCTTACTC.

### Enzyme-Linked Immunosorbent Assay (ELISA)

We performed ELISA to detect c-responsive protein (CRP) and rheumatic factor (RF) in serum and cytokines (TNF-α, IL-6, IL-1β, and IL-17) in the culture supernatant of cells, based on protocols of the ELISA kit (R&D Systems, USA; Yanhui Biological Reagent Co., China). We detected the erythrocyte sedimentation rate (ESR) according to the Westergren method.

### Western Blot

Proteins in pTHP-1 cells were purified by use of RIPA buffer (Beyotime, Shanghai, China). And the protease and phosphates inhibitors (Beyotime, Shanghai, China) were also used for protein isolation. A total of 30 μg proteins plus loading buffer were separated by gel electrophoresis. Specific monoclonal antibodies of TLR2, TLR4 (Santa Cruz Biotechnology, CA, USA), p-IκBα, p-NF-κB, and NF-κB (CST, USA) were adopted to capture proteins. The expression of specific proteins was normalized to β-actin (CST, USA) with three replicates.

### Cell Proliferation Assay

In this study, we used cell counting kit-8 (CCK-8) to detect cell proliferation at 24, 48, and 72 h by reagent kits (Sigma, USA). Cells were treated by the use of CCK-8 reagent solution, and then used for subsequent absorption determination. EdU was also performed to estimate the cell proliferation as previously reported (16).

### Fluorescence *in situ* Hybridization (FISH) Assay

After crawling, pTHP-1 cells were fixed with 4% paraformaldehyde for 10 min and then incubated with protease-K at 37°C for another 10 min. After washing with PBS, cells were gradient dehydrated with ethanol of different concentrations. Fluorescent labeled HIX003209 probe was used for hybridization. DAPI solution (Beyotime Biotechnology, Shanghai, China) was applied to nucleus staining.

### Immunofluorescence

The nuclear translocation of p-NF-κB in cells was determined by confocal laser scanning microscope after incubating with p-NF-κB monoclonal antibody (CST, USA). Nucleus was stained with DAPI solution (Beyotime Biotechnology, Shanghai, China).

### RNA Binding Protein Immunoprecipitation (RIP) Assay

RIP assay was carried out according to the protocol of Magna RIP RNA-Binding Protein Immunoprecipitation Kit (Millipore, Bedford, MA, USA). Cell lysate was incubated with RIP immunoprecipitation buffer containing magnetic beads, which could conjugate with TLR2, TLR4 (Abcam, Cambridge, USA), NF-κB (CST, USA), and IgG control antibody (Abcam, Cambridge, USA). HIX003209 RNA level in immunoprecipitates was determined by real-time PCR.

### Statistical Analysis

We applied the *T*-test or one-way ANOVA to estimate the data. A two-sided *P* < 0.05 was significant. In this study, SPSS (16.0v) and Graphpad (5.0v) softwares were used for statistical analysis.

## Results

### Increased Expression of lncRNA HIX003209 in RA

**Figure 1:**
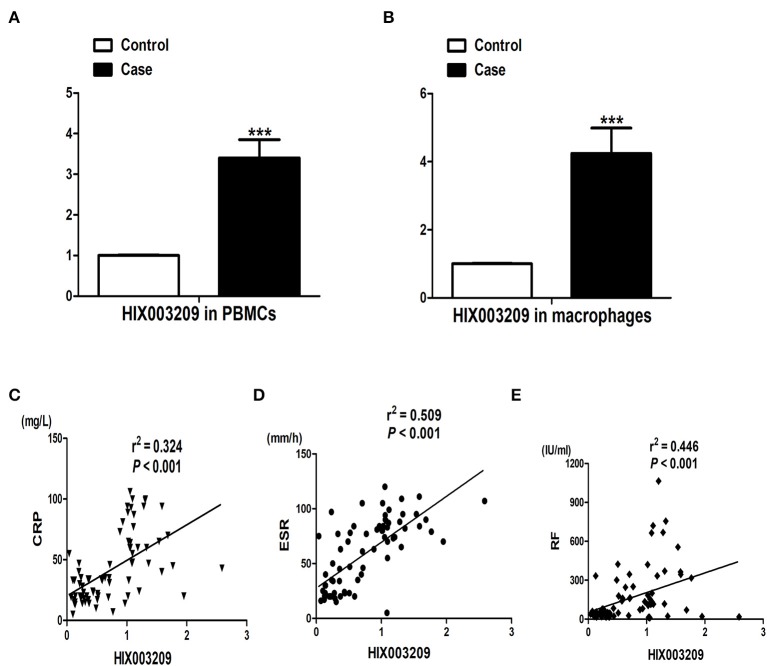
Expression of lncRNA HIX003209 and its association with disease activity in RA. **(A)** LncRNA HIX003209 expression in PBMCs samples from patients with RA in contrast to controls (patients/controls: 76/60; ***P < 0.001). **(B)** LncRNA HIX003209 expression in primary CD14^+^ mononuclear macrophages from RA patients and controls (patients/controls: 36/30; ***P < 0.001). **(C)** Positive association of lncRNA HIX003209 with CRP in RA (76 RA patients). **(D)** Positive association of lncRNA HIX003209 with ESR in RA (76 RA patients). **(E)** Positive association of lncRNA HIX003209 with RF in RA (76 RA patients).

We have found increased expression of lncRNA HIX003209 in serum from RA patients in a previous study (15). Similarly, elevated expression of lncRNA HIX003209 was observed in PBMCs and primary CD14^+^ macrophages from patients with RA (Figures 1A,B). Besides, positive association between the expression of lncRNA HIX003209 in PBMCs and CRP, ESR, and RF was identified in RA patients, respectively (Figures 1C–E). Taken together, lncRNA HIX003209 was up-regulated in RA and positively related to the disease activity.

### Association Between lncRNA HIX003209 and TLR2 and TLR4

**Figure 2:**
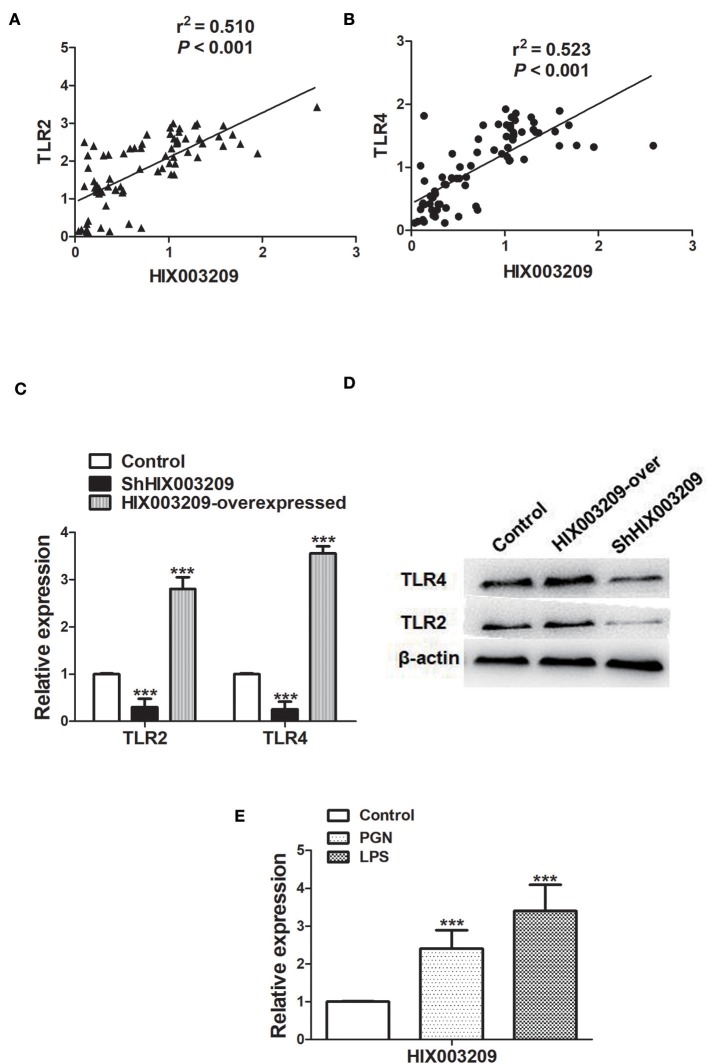
Association of lncRNA HIX003209 with TLR2 and TLR4 in RA. **(A)** Expression of lncRNA HIX003209 was positively associated with TLR2 in PBMCs of RA patients (76 cases). **(B)** Expression of lncRNA HIX003209 was positively associated with TLR4 in PBMCs of RA patients (76 cases). **(C)** Decreased TLR2 and TLR4 mRNAs in HIX003209 knockdown (shHIX003209) pTHP-1 cells while increased TLR2 and TLR4 mRNAs in HIX003209-overexpressed pTHP-1 cells (***P < 0.001; *n* = 3). **(D)** Decreased TLR2 and TLR4 proteins in shHIX003209 pTHP-1 cells while increased TLR2 and TLR4 proteins in HIX003209-overexpressed pTHP-1 cells. **(E)** Increased expression of HIX003209 in pTHP-1 cells when stimulated by PGN and LPS (***P < 0.001; *n* = 3) (Representative pictures of three independent experiments).

As shown in Figures 2A,B, the expression of lncRNA HIX003209 was positively correlated with TLR2 and TLR4 in RA. To further elucidate their relationship, the expression of lncRNA HIX003209 was knocked down with lentivirus shHIX003209 in pTHP-1 cells. The mRNA level of TLR2 and TLR4 was significantly reduced in HIX003209 knockdown macrophages compared with the control group (Figure 2C). Similarly, decreased expression of TLR2 and TLR4 proteins was also confirmed in HIX003209 knockdown pTHP-1 cells (Figure 2D) (Details were shown in Supplementary Material). However, over-expression of lncRNA HIX003209 promoted the expression of TLR2 and TLR4 in pTHP-1 cells (Figures 2C,D). Peptidoglycan (PGN) and lipopolysaccharide (LPS) were ligands for TLR2 and TLR4, respectively. When pTHP-1 macrophages were stimulated by PGN or LPS for 12 h, the expression of lncRNA HIX003209 was obviously enhanced as evidenced by real-time PCR (Figure 2E). Accordingly, TLR ligands (PGN and LPS) promoted the expression of lncRNA HIX003209 in pTHP-1 cells. Taken together, inflammatory stimuli enhanced the expression of lncRNA HIX003209 and thus further exaggerate the inflammatory response in macrophages.

### LncRNA HIX003209 Promoted Cell Proliferation and Activation Through IκBα/NF-κB Pathway

**Figure 3:**
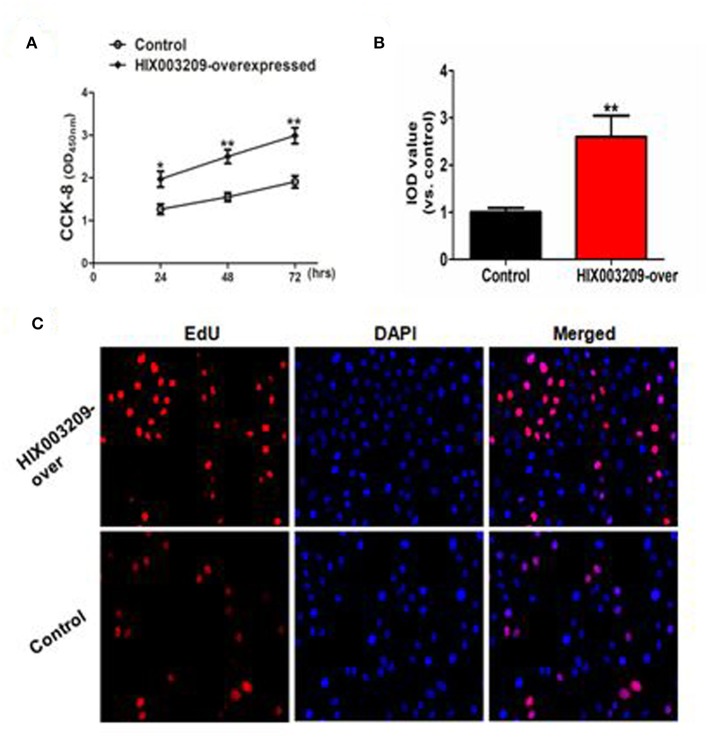
LncRNA HIX003209 promoted the proliferation of macrophages. **(A)** As demonstrated by CCK-8, the cell proliferation was enhanced in HIX003209-overexpressed cells (*P < 0.05; **P < 0.01; *n* = 3). **(B)** The integrated optical density (IOD) estimating pTHP-1 macrophages proliferation (**P < 0.01; *n* = 3). **(C)** As demonstrated by EdU the cell proliferation was significantly promoted when HIX003209 was over-expressed in cells (Representative pictures of three independent tests).

**Figure 4:**
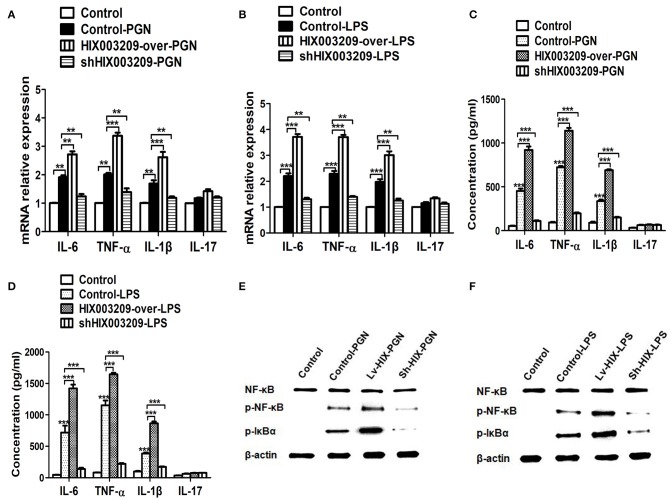
LncRNA HIX003209 enhanced the activation of macrophages through IκBα/NF-κB signaling pathway. **(A)** Expression of cytokines (IL-6, TNF-α, IL-1β, and IL-17) mRNAs was increased in HIX003209-overexpressed cells but decreased in shHIX003209 pTHP-1 cells despite the stimulation of PGN (**P < 0.01; ***P < 0.001; *n* = 3). **(B)** Expression of cytokines mRNAs was increased in HIX003209-overexpressed cells but decreased in shHIX003209 pTHP-1 cells although stimulated by LPS (**P < 0.01; ***P < 0.001; *n* = 3). **(C)** The production of cytokines in the supernatant of pTHP-1 cells stimulated by PGN was promoted when HIX003209 was over-expressed, but it was inhibited when HIX003209 was knocked down in cells (***P < 0.001; *n* = 3). **(D)** The generation of cytokines in the supernatant of pTHP-1 cells stimulated by LPS was enhanced when HIX003209 was over-expressed, but it was restrained when HIX003209 was knocked down in cells (***P < 0.001; *n* = 3). **(E,F)** Western blot analysis showed increased phosphorylation and activation of IκBα/NF-κB in macrophages with HIX003209 overexpression (*n* = 3, representative pictures).

**Figure 5:**
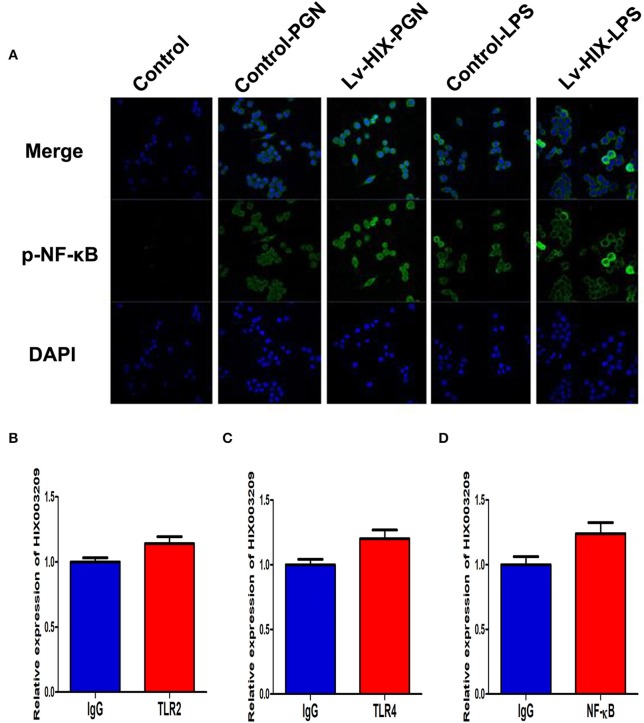
LncRNA HIX003209 activated NF-κB signaling but not directly bound to TLR2, TLR4, and NF-κB in macrophages. **(A)** Immunofluorescence demonstrated increased activation and nuclear translocation of p-NF-κB in macrophages with HIX003209 upregulation (representative figures of three repeated tests). **(B)** RIP showed expression level of HIX003209 in immunoprecipitates as fold enrichment of TLR2 relative to IgG determined by real-time PCR (*n* = 3). **(C)** Expression of HIX003209 in immunoprecipitates as fold enrichment of TLR4 relative to IgG (*n* = 3). **(D)** HIX003209 expression in immunoprecipitates from macrophages extracts as fold enrichment of NF-κB relative to IgG (*n* = 3).

As assayed by cell proliferation assays (CCK-8 and EdU), over-expression of lncRNA HIX003209 could promote cell proliferation (Figures 3A–C). Increased levels of TNF-α, IL-6 and IL-1β mRNAs were found in PGN- and LPS-stimulated pTHP-1 macrophages (Figures 4A,B). Besides, the generation of TNF-α, IL-6, and IL-1β mRNAs was significantly promoted in lncRNA HIX003209-overexpressed macrophages stimulated by PGN and LPS (Figures 4A,B). Similarly, proteins of TNF-α, IL-6, and IL-1β were obviously increased in the cultural supernatant of PGN- and LPS-stimulated lncRNA HIX003209-overexpressed macrophages (Figures 4C,D). Nevertheless, obviously reduced levels of inflammatory cytokines (TNF-α, IL-6, and IL-1β) were observed in lncRNA HIX003209 knockdown pTHP-1 cells in spite of the stimulation of PGN and LPS (Figures 4A–D). There was no statistical difference for IL-17 between groups (Figures 4A–D). Moreover, lncRNA HIX003209 promoted the production of inflammatory cytokines in macrophages depending on the activation of IκBα/NF-κB signaling pathway (Figures 4E,F, 5A). Taken together, lncRNA HIX003209 could enhance the proliferation and activation of macrophages through TLR/NF-κB pathway. Given this, we hypothesized whether HIX003209 could bind directly to these proteins to display its regulatory role in macrophages. Unfortunately, we found that lncRNA HIX003209 could not directly bind to TLR2, TLR4, and NF-κB, suggesting RNA binding protein immunoprecipitation (Figures 5B–D).

### MiR-6089 Was a Target of lncRNA HIX003209

**Figure 6:**
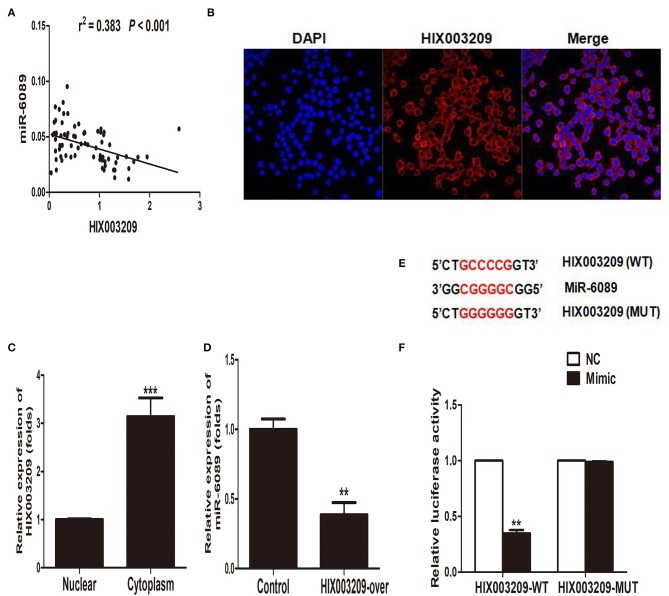
MiR-6089 was a targeted gene of lncRNA HIX003209. **(A)** The expression of LncRNA HIX003209 was negatively associated with miR-6089 in PBMCs from RA (76 cases). **(B)** FISH assay showed lncRNA HIX003209 was primarily expressed in cytoplasm of macrophages (one representative figure from three repeated tests). **(C)** Real-time PCR detected the expression of lncRNA HIX003209 in macrophages (***P < 0.001; *n* = 3). **(D)** HIX003209 inhibited the expression of miR-6089 in macrophages (**P < 0.01; *n* = 3). **(E)** Complementary pairing bases of HIX003209 and miR-6089 (WT, wild type; MUT, mutant). **(F)** Luciferase reporter assay demonstrated HIX003209 could targetedly regulate miR-6089 (**P < 0.01; *n* = 3).

In our previously study, miR-6089 was found to play an important role in RA pathogenesis by targeting TLR4 (16). In this study, we had found a positive association between lncRNA HIX003209 and TLR4 with regard to their expression in RA (Figure 2B), and the modifying effect of LPS/TLR4-mediated inflammation in macrophages. As a result, we hypothesized that lncRNA HIX003209 might affect inflammatory response by regulating miR-6089/TLR4. Interestingly, it was demonstrated that lncRNA HIX003209 was negatively related to miR-6089 regarding the expression in PBMCs samples of RA cases (Figure 6A). LncRNA HIX003209 was primarily expressed in the cytoplasm of pTHP-1 cells (Figures 6B,C). The expression of miR-6089 was significantly decreased when HIX003209 was over-expressed in macrophages (Figure 6D). Accordingly, lncRNA HIX003209 might function as a ceRNA by sponging miR-6089 in pTHP-1 macrophages. There were six complementary pairing bases between HIX003209 and miR-6089 (Figure 6E). Furthermore, the luciferase reporter assay showed that HIX003209 could specifically recognize miR-6089 (Figure 6F). Taken together, miR-6089 was a direct target of lncRNA HIX003209. LncRNA HIX003209 could sponge miR-6089 as a ceRNA.

### LncRNA HIX003209 Influenced the Downstream Signaling of miR-6089/TLR4 in Macrophages Via NF-κB

**Figure 7:**
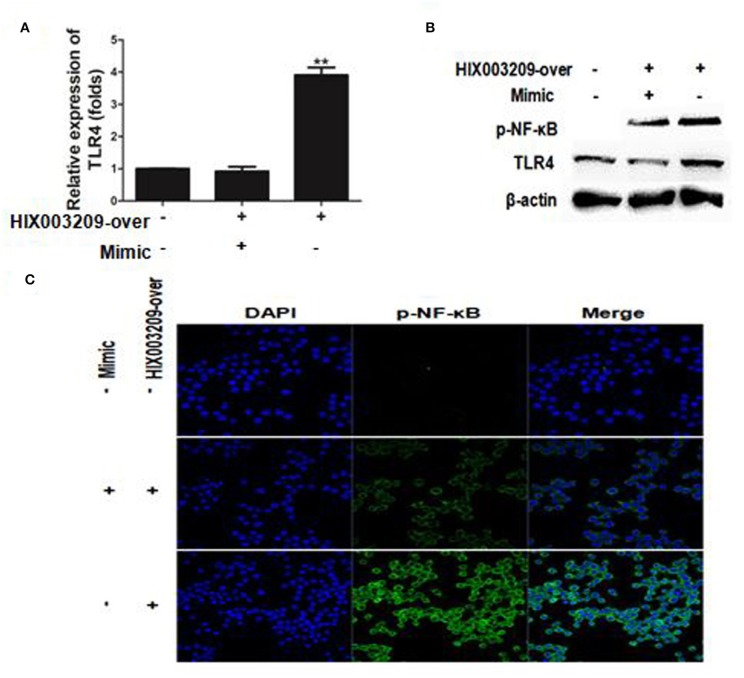
LncRNA HIX003209 functioned as a ceRNA by sponging miR-6089 through TLR4/NF-κB signaling in macrophages. **(A)** LncRNA HIX003209 promoted the expression of TLR4 by sponging miR-6089 in macrophages (**P < 0.01; *n* = 3). **(B)** LncRNA HIX003209 enhanced the expression of TLR4 and the phosphorylation of NF-κB by sponging with miR-6089 (*n* = 3, representative pictures). **(C)** LncRNA HIX003209 boosted the nuclear translocation of p-NF-κB in macrophages by sponging with miR-6089 (*n* = 3, representative pictures).

The regulatory mechanism of HIX003209 in RA pathogenesis is not yet clear. Here, lncRNA HIX003209 was shown to promote the expression of TLR4 by functioning as a ceRNA and sponging miR-6089, while mimics of miR-6089 could inhibit the expression of TLR4, although HIX003209 was over-expressed in cells (Figures 7A,B) (Details were shown in Supplementary Material). Besides, lncRNA HIX003209 enhanced the activation of NF-κB with a high level of phosphorylation and increased nuclear translocation in macrophages (Figures 7B,C). However, mimics of miR-6089 could restrain phosphorylation and nuclear translocation of NF-κB in macrophages. Taken together, lncRNA HIX003209 acted as a ceRNA and regulated miR-6089/TLR4 through NF-κB signaling in macrophages.

## Discussion

The current study firstly provides evidence that lncRNA HIX003209 is involved in the pathogenesis of RA by enhancing macrophage-mediated inflammatory response via TLR2/TLR4. LncRNA HIX003209 enhances macrophages proliferation and activation through IκBα/NF-κB signaling pathway. Most importantly, lncRNA HIX003209 can function as a ceRNA by effectively binding to miR-6089, which restores the expression of TLR4 and the activation of downstream signaling molecule NF-κB in macrophages. The lncRNA HIX003209-miR6089-TLR4 network offers promising therapeutic strategy for RA patients.

LncRNAs have more than 200 nucleotides in length, which possess capacities of regulating a variety of coding genes (17). It has been well-established that lncRNAs play important roles in the regulation of autoimmunity and inflammatory response (18–20). Dysregulation of lncRNAs in lymphocytes is established to be involved in the immunopathogenesis of rheumatoid diseases, including systematic lupus erythematosus (SLE) and RA (13, 19, 21). Apart from directly binding to proteins, some lncRNAs can indirectly regulate mRNAs by sponging miRNAs. During the past few years, lncRNA-miRNA-mRNA ceRNA theory has been demonstrated in the development of multiple diseases, such as malignancies, cardiovascular diseases and autoimmune diseases (21–24). LncRNAs are capable of acting as miRNA decoys to restore the expression of targeted genes via competitive regulatory interactions between lncRNAs, miRNAs, and mRNAs. Aberrant expression of any non-coding RNAs in this network would contribute to the occurrence and progression of certain diseases. A recent study by Jiang and the colleagues has revealed functional lncRNAs in RA based on the ceRNA theory (13). There are a few lncRNAs that have been demonstrated to affect the proliferation, invasion, and migration of fibroblast-like synoviocytes in RA by suppressing miRNAs via ceRNA network, such as GAPLINC (21) and ZFAS1 (25). Nevertheless, the molecular mechanisms of well-established lncRNAs as miRNAs sponge in RA still need to be further elucidated, which will facilitate the identification of valuable and effective targets for RA diagnosis and treatment based on the lncRNA-miRNA-mRNA ceRNA network. Our study firstly provides evidence that lncRNA HIX003209 is dysregulated in macrophages, and promotes the proliferation and inflammatory cytokines (TNF-α, IL-6, and IL-1β) generation of macrophages through IκBα/NF-κB pathway. LncRNA HIX003209 cannot directly bind to TLR2, TLR4, and the downstream protein NF-κB, but it can sponge miR-6089 and further promotes the expression of TLR4/NF-κB in macrophages through ceRNA mechanism. Knockdown of lncRNA HIX003209 can alleviate inflammation in macrophages. Accordingly, the newly identified lncRNA HIX003209-miR6089-TLR4 ceRNA network will provide new insight into understanding the pathogenesis of RA. Novel targets for RA treatment require further investigation in future studies based on this ceRNA network.

Toll-like receptors (TLRs) and TLRs-mediated signaling transduction are closely associated with inflammation, tumors and autoimmune regulations (26, 27). TLRs and its downstream signaling pathways, such as MAPK, Wnt, and NF-κB pathways, have been elucidated in synovial inflammation and bone remodeling of RA (28–30). Previously, we have found that TLR4-mediated innate immune and inflammatory response play a vital role in RA, primarily depending on NF-κB signaling activation (16, 31, 32). TLR2-mediated immune and inflammatory response also play important roles in RA (33, 34). Taken together, TLRs confer significant effects on the pathogenesis of RA. It has been well-documented that many non-coding RNAs can targetedly regulate specific TLRs, and thus contribute to the development of RA, including lncRNAs (15, 16, 30). Many studies have implicated the critical role of lncRNAs in regulating autoimmune and inflammation by targeting TLRs, such as TLR2, TLR4, and TLR3 (35–37). Most interestingly, more and more published studies have suggested that some established lncRNAs can regulate TLR signaling transduction and the relevant immune function in cancer, autoimmune and inflammatory disorders by acting as ceRNAs, such as networks of lncRNA SNHG1-miR-140-TLR4, lncRNA X-miR-154-5p-TLR5, and lncRNA Gm6135-miR-203-3p-TLR4 (38–41). However, no available data can support the interaction between lncRNA and miRNA in regulating TLR signaling pathway in RA up to date. In this study, we have found lncRNA HIX003209 contributes to RA by regulating TLR2- and TLR4-mediated inflammation in macrophages. Most importantly, lncRNA HIX003209 is capable of restoring the expression of TLR4 and activation of NF-κB by sponging miR-6089 in macrophages. As a result, HIX003209 can function as a ceRNA and regulate TLR4/NF-κB signaling pathway via targeting miR-6089 in RA. However, future studies are warranted to identify more promising targets in the network of HIX003209-miR-6089-TLR4, particularly in the downstream of TLR4 signaling.

Inflammatory cells and inflammatory mediators play crucial roles in soft tissue injuries and bone lesions in RA, such as IL-6, TNF-α, and IL-1β. Increased inflammatory cytokines result in infiltration of macrophages and progressive destruction of articular cartilage, and ultimately bone (42, 43). Accordingly, it is useful for treatment by blocking inflammation-associated molecules and pathways involved in RA. Certain inhibitors of inflammatory cytokines have been applied into clinical treatments of RA, such as IL-6R monoclonal antibody and TNF-α inhibitors. Researchers have attempted to explore novel strategies for RA treatment by inhibiting NF-κB signaling pathway, a key pathway regulating inflammation (44, 45). In the present study, we have elucidated that lncRNA HIX003209 promotes PGN- and LPS-induced inflammatory response via TLR2 and TLR4 signaling pathway in macrophages. Knockdown of lncRNA HIX003209 helps to alleviate inflammation in macrophages. As a result, shHIX003209 may be a useful reagent for the treatment of RA. However, more research is warranted to explore a useful strategy for RA targeted treatment by blocking any node in the HIX003209-miR-6089-TLR4 network, particularly experiments *in vivo*.

To summarize, the study firstly demonstrates the altering effect of lncRNA HIX003209 in RA by regulating macrophages-mediated inflammation. The HIX003209-miR-6089-TLR4 network provides novel therapeutic targets for RA patients in future.

## Data Availability

All datasets generated for this study are included in the manuscript/Supplementary Files.

## Ethics Statement

Written informed consent was obtained from the individual(s) for the publication of any potentially identifiable images or data included in this article.

## Author Contributions

SY, MC, and DX designed the experiments. SY, PW, JW, and JY carried out the experiments. CJ and HL gave advice on experimental design and data analysis. SY, PW, and JW wrote and revised the paper. MC and DX edited the article.

### Conflict of Interest Statement

The authors declare that the research was conducted in the absence of any commercial or financial relationships that could be construed as a potential conflict of interest.
