# Supplementary figures and images for: Long Non-coding RNA HIX003209 Promotes Inflammation by Sponging miR-6089 via TLR4/NF-κB Signaling Pathway in Rheumatoid Arthritis
Source: Front Immunol. 2019 Sep 18;10:2218. doi: 10.3389/fimmu.2019.02218 (PMC6759987; doi:10.3389/fimmu.2019.02218)

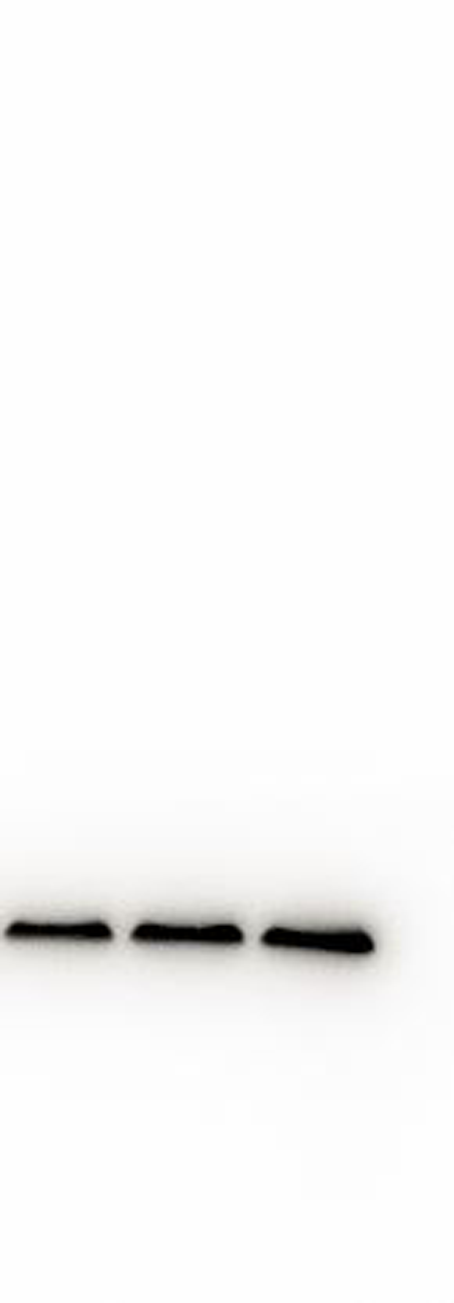

Supplement: Supplementary file 1 [file Data_Sheet_1.ZIP › Actin Figure 2D.tif]

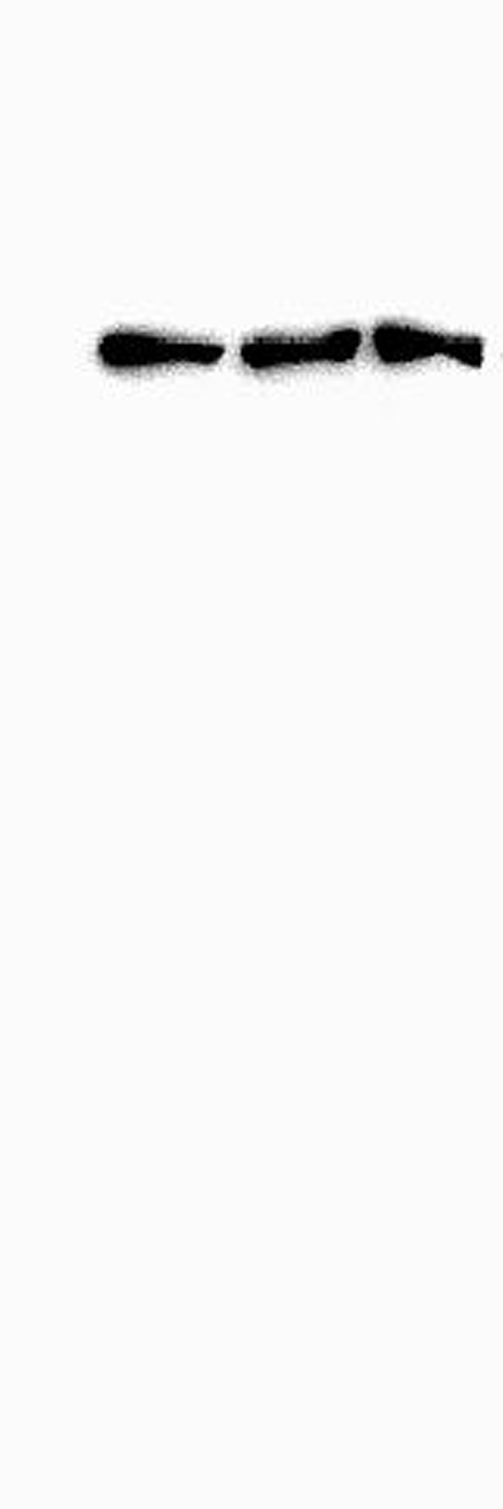

Supplement: Supplementary file 1 [file Data_Sheet_1.ZIP › Actin Figure 6B.tif]

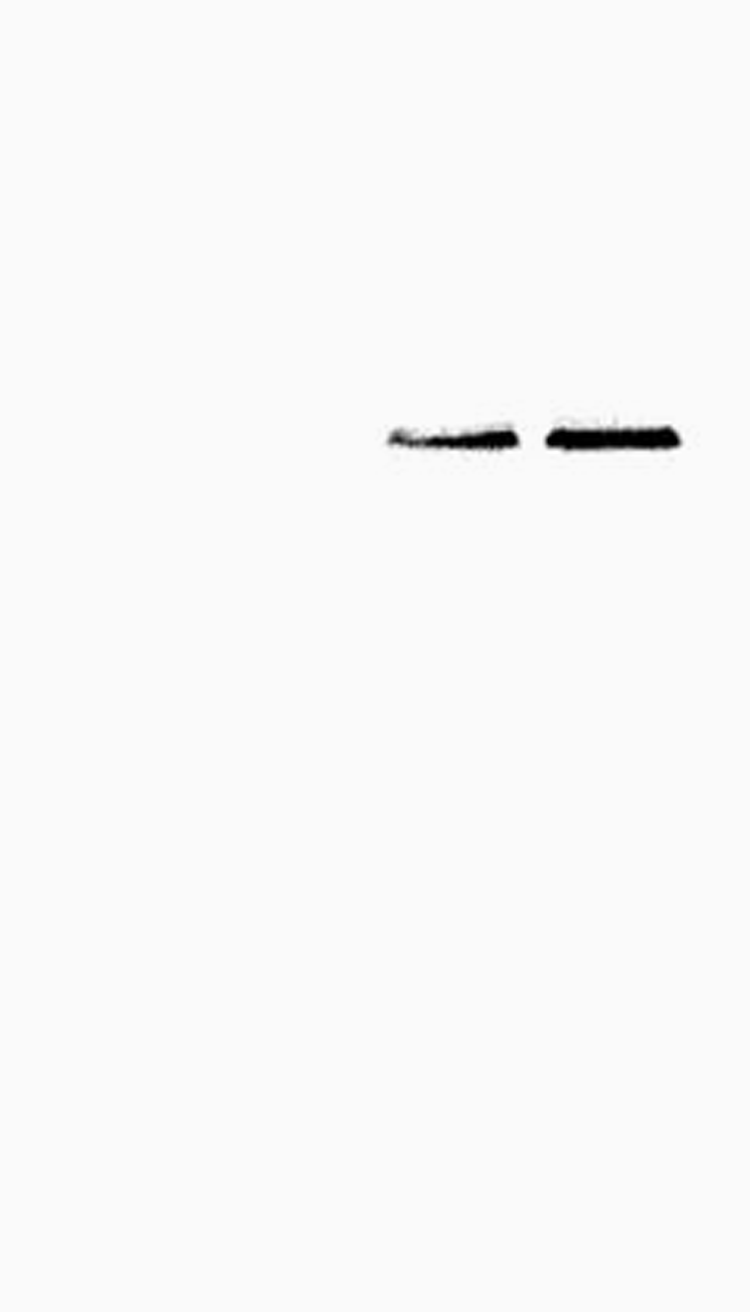

Supplement: Supplementary file 1 [file Data_Sheet_1.ZIP › NFkB Fig6B.tif]

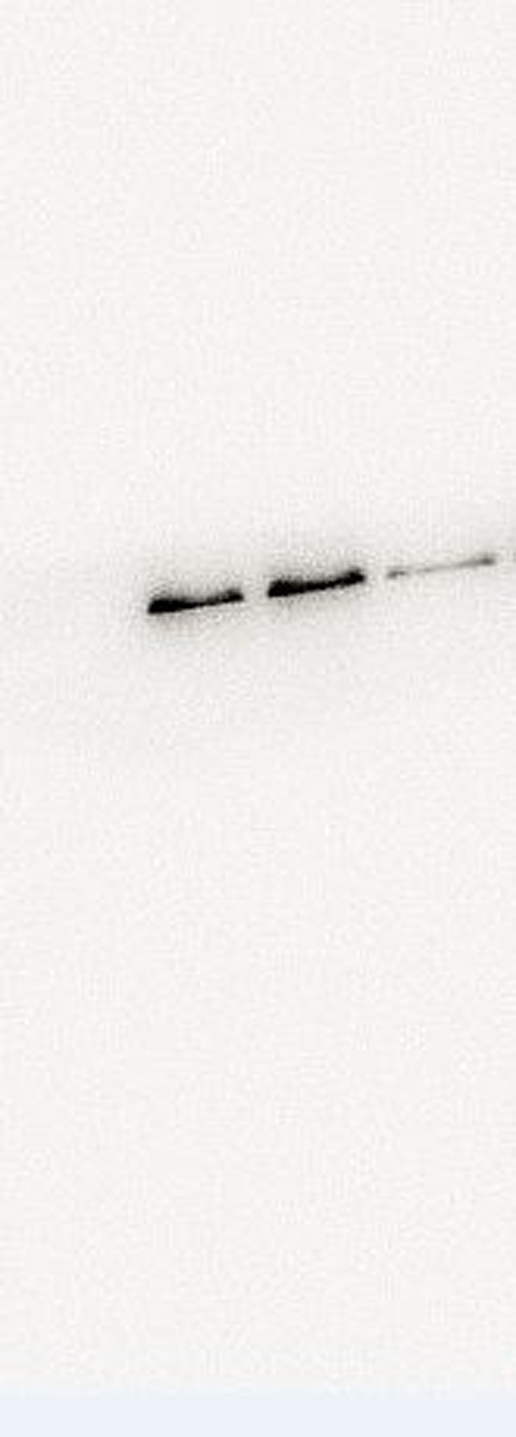

Supplement: Supplementary file 1 [file Data_Sheet_1.ZIP › TLR2 Fig2D.tif]

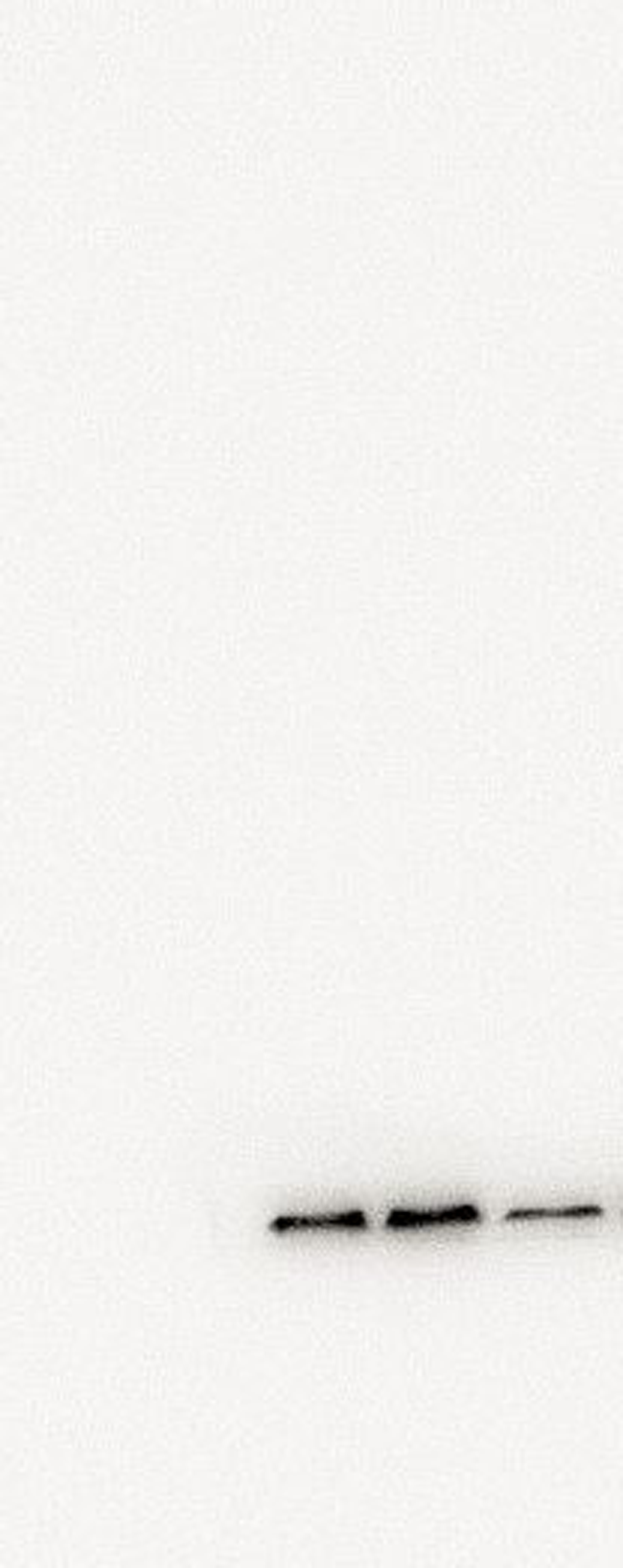

Supplement: Supplementary file 1 [file Data_Sheet_1.ZIP › TLR4 Fig2D.tif]

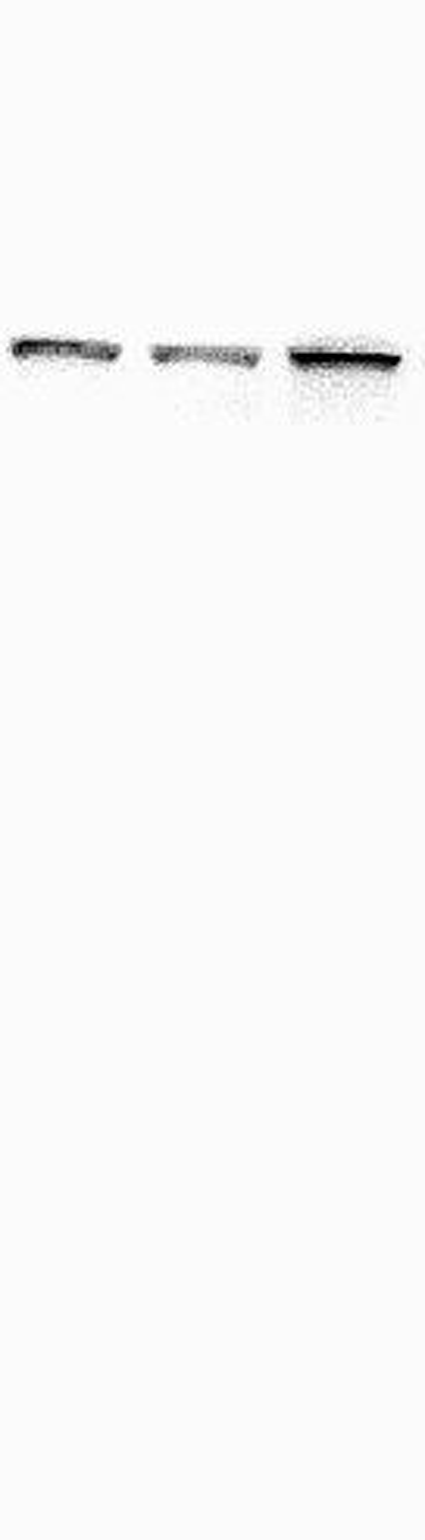

Supplement: Supplementary file 1 [file Data_Sheet_1.ZIP › TLR4 Fig6B.tif]
